# OrgNet: orientation-gnostic protein stability assessment using convolutional neural networks

**DOI:** 10.1093/bioinformatics/btaf252

**Published:** 2025-07-15

**Authors:** Ilya Buyanov, Anastasia Sarycheva, Petr Popov

**Affiliations:** iMolecule, Skolkovo Institute of Science and Technology, Moscow 121205, Russia; Constructor Knowledge Labs, Bremen 28759, Germany; Constructor University Bremen gGmbH, Bremen 28759, Germany; Tetra D AG, Sсhaffhausen 8200, Switzerland; Constructor Knowledge Labs, Bremen 28759, Germany; Constructor University Bremen gGmbH, Bremen 28759, Germany; Tetra D AG, Sсhaffhausen 8200, Switzerland

## Abstract

**Motivation:**

Accurately predicting the impact of single-point mutations on protein stability is crucial for elucidating molecular mechanisms underlying diseases in life sciences and advancing protein engineering in biotechnology. With recent advances in deep learning and protein structure prediction, deep learning approaches are expected to surpass existing methods for predicting protein thermostability. However, structure-based deep learning models, specifically convolutional neural networks, are affected by orientation biases, leading to inconsistent predictions with respect to the input protein orientation.

**Results:**

In this study, we present OrgNet, a novel orientation-gnostic deep learning model using 3D convolutional neural networks to predict protein thermostability change upon point mutation. OrgNet encodes protein structures as voxel grids, enabling the model to capture fine-grained, spatially localized atomic features. OrgNet implements spatial transforms to standardize input protein orientations, thus eliminating orientation bias problem. When evaluated on established benchmarks, including $S^{\mathrm{sym}}$ and $S^{669}$, OrgNet achieves state-of-the-art performance, demonstrating superior accuracy and robust performance compared to existing methods.

**Availability and implementation:**

OrgNet is available at https://github.com/i-Molecule/OrgNet.

## 1 Introduction

Protein thermodynamic stability, often characterized by the protein folding free energy change (Δ*G*), is a fundamental parameter in understanding protein properties and function (Aldeghi *et al.* 2019). Generally, Δ*G* depends on intramolecular interactions between amino acid residues of the protein and the intermolecular interactions between the protein and its surrounding environment (Honig and Yang 1995). Mutations that alter amino acid residues can influence protein stability by either stabilizing or destabilizing the mutant protein relative to the wild-type (WT) protein (Tokuriki and Tawfik 2009). The impact of a mutation on protein stability is quantified by the change in folding free energy upon mutation (ΔΔ*G*), a parameter of critical importance in medicine, protein design, and biotechnology (Sanavia *et al.* 2020). Many pathogenic mutations, such as single-point amino acid substitutions, can significantly destabilize proteins, contributing to various molecular disorders (Clausen *et al.* 2019). Predicting the effects of genetic variations on protein function and stability is a key focus of large-scale genetic studies. In biopharmaceuticals, designing proteins with specific thermodynamic properties is a major objective (Frokjaer and Otzen 2005). However, experimental screening of numerous protein mutants is labor-intensive and time-consuming. Consequently, there is an unmet need for precise computational methods to prioritize mutant proteins for experimental validation. Therefore, various computational methods have been devised to estimate ΔΔG [we refer the reader to detailed reviews here (Sanavia *et al.* 2020, Pancotti *et al.* 2022, Qiu *et al.* 2024)]. These methods can be broadly classified into three categories: empirical approaches, free energy perturbation (FEP) protocols, and machine learning or statistical learning models. Empirical approaches, such as Rosetta (Das and Baker 2008), FoldX (Schymkowitz *et al.* 2005), or Eris (Yin *et al.* 2007), leverage force fields to model the impact of mutations on protein stability. While these methods are suitable for large-scale computations, their accuracy is often limited by the inherent simplifications and approximations in their scoring functions (Potapov *et al.* 2009). On the other hand, the FEP protocols, that estimate free energy changes caused by point mutations using molecular dynamics simulations, offer higher accuracy, but are computationally demanding (Seeliger and De Groot 2010). The low throughput of such physics-based approaches limits their scalability, making them less suitable for high-throughput protein engineering and variant interpretation tasks. Nowadays, machine learning methods represent a powerful alternative and are well-suited for large-scale applications in protein stability prediction. Several methods based on classical machine learning algorithms, such as Random Forest [e.g. DynaMut2 (Rodrigues *et al.* 2021)] and XGBoost [e.g. iStable 2.0 (Chen *et al.* 2020a)], are widely used in this domain (Horne and Shukla (2022), Pancotti *et al.* (2022)). Machine learning methods are typically classified into sequence-based and structure-based approaches; however, the most recent methods tend to utilize diverse descriptors to improve predictive performance. For instance, DDGun3D (Montanucci *et al.* 2022) or PremPS (Chen *et al.* 2020b) combine evolutionary, statistical, and structure-based data. The rapid development of deep learning offers the potential to further enhance the predictive performance of protein stability predictors. Particularly, inspired by advances in large language models, methods based on protein language models (PLMs) have demonstrated promising results (Gong *et al.* 2023, Umerenkov *et al.* 2023). On the other hand, ThermoNet (Li *et al.* 2020) utilized grid representation of a mutation site, which is processed with a 3D convolutional neural network (CNN) similar to powerful 2D CNN approaches in computer vision.

Notably, 3D CNNs have shown their efficacy in addressing diverse structural biology problems, such as virtual ligand screening (McNutt *et al.* 2021) and *de novo* drug design (Francoeur *et al.* 2020). However, a significant limitation of 3D CNNs is their lack of SE(3) invariance, meaning that the output prediction depends on the input’s orientation. This poses a challenge for 3D CNN-based protein thermostability prediction methods, as the predictions for a given point mutation should be consistent regardless of the protein’s orientation in 3D space. Moreover, this SE(3)-variance can compromise the robustness of such methods, potentially leading to discrepancies in predictions, including differences in the sign of the ΔΔ*G* value for different orientations of the same protein. To the best of our knowledge, no protein thermostability prediction method leverages 3D CNNs while ensuring robustness with respect to the protein orientation. In this study, we present OrgNet, a robust 3D CNN-based method for predicting ΔΔG upon single-point mutations. Our approach represents a point mutation using two voxel grids, calculated for the wild-type (WT) and mutant-type (MT) structures, capturing the physicochemical properties of the atomic environment around the mutation site. To address the issue of SE(3)-variance, we introduced an orientation standardization, that applies spatial transformation to the input protein structure, ensuring that the computed voxel grids remain invariant to protein orientation. We show that this enhancement significantly improves the performance of 3D CNN-based models, such as ThermoNet (Li *et al.* 2020), and introduce an optimized 3D CNN architecture that outperforms or matches structure-based state-of-the-art methods.

## 2 Materials and methods

### 2.1 Datasets

The datasets for protein stability change upon point mutations typically contain information about ΔΔ*G*, which represents the difference between Gibbs free energy changes (Δ*G*) of the mutant and wild-type variants:


(1) $$\Delta \Delta G=\Delta G_{\mathrm{mutant}}-\Delta G_{\mathrm{wildtype}}$$

Thus, a destabilizing mutation corresponds to a positive value of ΔΔ*G*, while a stabilizing mutation corresponds to a negative value. For a set of point mutations, where amino acid residue $A_{i}$ is replaced with amino acid residue $B_{j}$: ($A_{i}\to B_{j}$) with associated value $\Delta \Delta G_{A_{i}\to B_{j}}$, these datasets can be augmented with inverse mutations $B_{j}\to A_{i}$ and their associated value $\Delta \Delta G_{B_{j}\to A_{i}}=-\Delta \Delta G_{A_{i}\to B_{j}}$. An accurate ΔΔ*G* predictor should provide consistent predictions for both direct and reverse mutations, a requirement often overlooked in previous works. In this study, we used (i) Q1744 and Q3214 (Li *et al.* 2020) as training and $S^{\mathrm{sym}}$ (Pucci *et al.* 2018) as test datasets for comparison with ThermoNet, and (ii) S2648 (Dehouck *et al.* 2011) as the main training set. We used 5-fold cross-validation using a homology-based split of the S2648 dataset (Savojardo *et al.* 2016) to train the final model. Specifically, similar proteins were determined using BLASTP (Sayers *et al.* 2022) and clustered together using connected components with an edge threshold of 25%. Subsequently, a random partition was generated that grouped proteins from the same cluster into the same set to produce homology-based cross-validation splits. This approach reduces potential biases arising from similarities between training and testing sets. For a fair evaluation of the methods, we used $S^{669}$ (Pancotti *et al.* 2022) as the test set, as it was specifically composed to share no similar proteins with S2648. Note, that we have augmented the datasets by creating a reverse mutation for each mutation. It is also important to note that there is overlap between the training datasets, with homologous sequences shared across them. We have calculated sequence-based similarity between the datasets using various similarity thresholds (see Supplementary Material, Supplementary Fig. S2 and Supplementary Table S3, for more details).

To generate the augmented dataset, we created a grid of uniformly distributed unit axes (Morozov *et al.* 2022) and uniformly distributed angles (ranging from 0 to 2π with a step size of π/36). Next, we randomly selected one axis and one angle ten times and computed the corresponding rotation matrices. Each rotation matrix was then applied to the centered coordinates of the protein, resulting in a new orientation. Thus, for each protein structure, we added ten random orientations to the dataset. To generate flexible deformations of the protein structures, we used molecular dynamics simulations with GROMACS (Van Der Spoel *et al.* 2005). Specifically, we prepared a simulation box containing the protein, water molecules, and NaCl ions at a concentration of 0.15M, with a 2 nm margin from any atom of the protein. We followed the standard procedure of energy minimization, NVT and NPT equilibration for 1 ns, and a production run of 100 ns using the amber14sb force field (Maier *et al.* 2015). Finally, we retrieved 100 conformations (one per ns), resulting in the final conformational ensemble.

### 2.2 Point mutation representation

For each record in a dataset, we retrieved the wild-type protein structure from PDB (Berman *et al.* 2000), modeled the mutant-type structure using Rosetta (Kellogg *et al.* 2011), and relaxed both structures using the Rosetta FastRelax protocol with backbone constraints (Tyka *et al.* 2011). We then processed each structure using the cubic map positioning approach (Pagès *et al.* 2019). Given an amino acid residue *k* of interest, we determined three basis vectors x, y, and z as follows. The x vector corresponds to the vector from the carbon atom of the preceding residue carboxyl group $C^{k-1}$ to the nitrogen atom of the current residue amino group $N^{k}$ (see Fig. 2A). The y vector is then chosen in the $C^{k-1}-N^{k}-C_{\alpha }^{k}$ plane to be perpendicular to x, such that the $C_{\alpha }^{k}$ atom lies in the half-plane Oxy with y>0 (see Fig. 2A). This orientation is achieved by subtracting from the $\vec{N^{k}C_{\alpha }^{k}}$ vector its projection onto the x axis. Finally, the z vector is defined as the cross product of x and y, and the original structure is oriented with respect to this new basis. For each structure, we computed a grid of 16×16×16 voxels centered at the $C_{\beta }$ atom. Similarly to ThermoNet, the voxel grid encodes seven channels representing hydrophobicity, hydrogen bond acceptor capacity, hydrogen bond donor capacity, aromaticity, positive ionizability, negative ionizability, and occupancy properties, calculated using the HTMD library (Doerr *et al.* 2016). Given voxel grids computed for the wild-type and the mutant-type structures ([WT] and [MT], respectively), we constructed the point mutation representation by combining these grids in one of three ways: [WT, MT], [WT, WT-MT], or [WT-MT]. Note, that for a few point mutations (listed in Supplementary Material, Supplementary Table S4), the pipeline failed to compute the representation; those point mutations were excluded from consideration.

### 2.3 Model training

To implement the ThermoNet-like model, we followed the protocol from Li *et al.* (2020). The ThermoNet architecture comprises three convolutional layers with sizes 16, 24, and 32, and a dense layer with size 24. ThermoNet processes input tensors with a shape of [16, 16, 16, 14] and contains 133 273 parameters. The hyperparameter values and loss function were also adopted from Li *et al.* (2020). Supplementary Table S1 demonstrates that the performance metrics of the reproduced ThermoNet and the original ThermoNet are similar. To construct the 3D Steerable ThermoNet-like model, we implemented the ThermoNet architecture in PyTorch, replacing conventional convolutional layers with 3D steerable convolutional layers introduced in Weiler *et al.* (2018) and available at the GitHub repository (https://github.com/bkmi/e3nnet.git). The hyperparameter values and loss function were also adopted from Li *et al.* (2020). To develop the final 3D CNN architecture for OrgNet, we explored various configurations of 3D convolutional blocks and the regression head using 5-fold cross-validation on homology-based splits of the S2648 dataset (Dehouck *et al.* 2009). Optimization was performed with the mean squared error (MSE) loss, and the best validation scores, averaged across folds, guided the selection of the final architecture, which is as follows. The input convolutional block consisted of a 3D convolutional layer with a kernel size of 3 (k=3), padding of 1 (p=1), and stride of 1 (s=1), producing 16 output channels. This was followed by a ReLU activation function and a 3D max-pooling layer with k=3, p=1, and s=2. Two intermediate convolutional blocks were included, each featuring a 3D convolutional layer (k=3, p=1, s=1) with 80 and 400 output channels, respectively. Each convolutional layer was followed by a ReLU activation function and a 3D max-pooling layer. The output block comprised a 3D convolutional layer (k=2, p=0, s=1) with 512 output channels, followed by a ReLU activation function. All convolutional and max-pooling layers used a dilation factor of 1. A flattened layer connected the output block to the regression head, consisting of two fully connected layers with sizes 512 and 128, each utilizing a GELU activation function, leading to the prediction. In total, the optimized architecture included 2 871 457 parameters. OrgNet was trained using the described architecture with the same 5-fold split for 100 epochs and a batch size of 8. The Adam optimizer was used with an initial learning rate of 0.001, and the ReduceLROnPlateau scheduler decreased the learning rate by a factor of 0.5 after 10 epochs of no improvement in validation loss, with a cooldown period of 5 epochs. Early stopping, based on validation loss, was implemented with a patience of 15 epochs. The best model from each fold was saved, and ensemble predictions were averaged. The final model achieved a mean training MSE of 1.33 ± 0.25 and a mean validation MSE of 1.36 ± 0.37. When retraining OrgNet on the Q3214 dataset using random-split 5-fold cross-validation, the ReduceLROnPlateau scheduler was adjusted to use a factor of 0.9, patience of 5 epochs, and no cooldown. This resulted in the ensemble model with a mean training MSE of 1.58 ± 0.42 and a mean validation MSE of 1.20 ± 0.80.

For performance evaluation, the Pearson correlation coefficient (r), root-mean-squared error (σ,RMSE), mean absolute error (MAE), and mean squared error (MSE) were calculated between the experimental and predicted ΔΔ*G* values.

## 3 Results

### 3.1 Impact of protein structure orientation on the stability prediction

**Figure 1.**
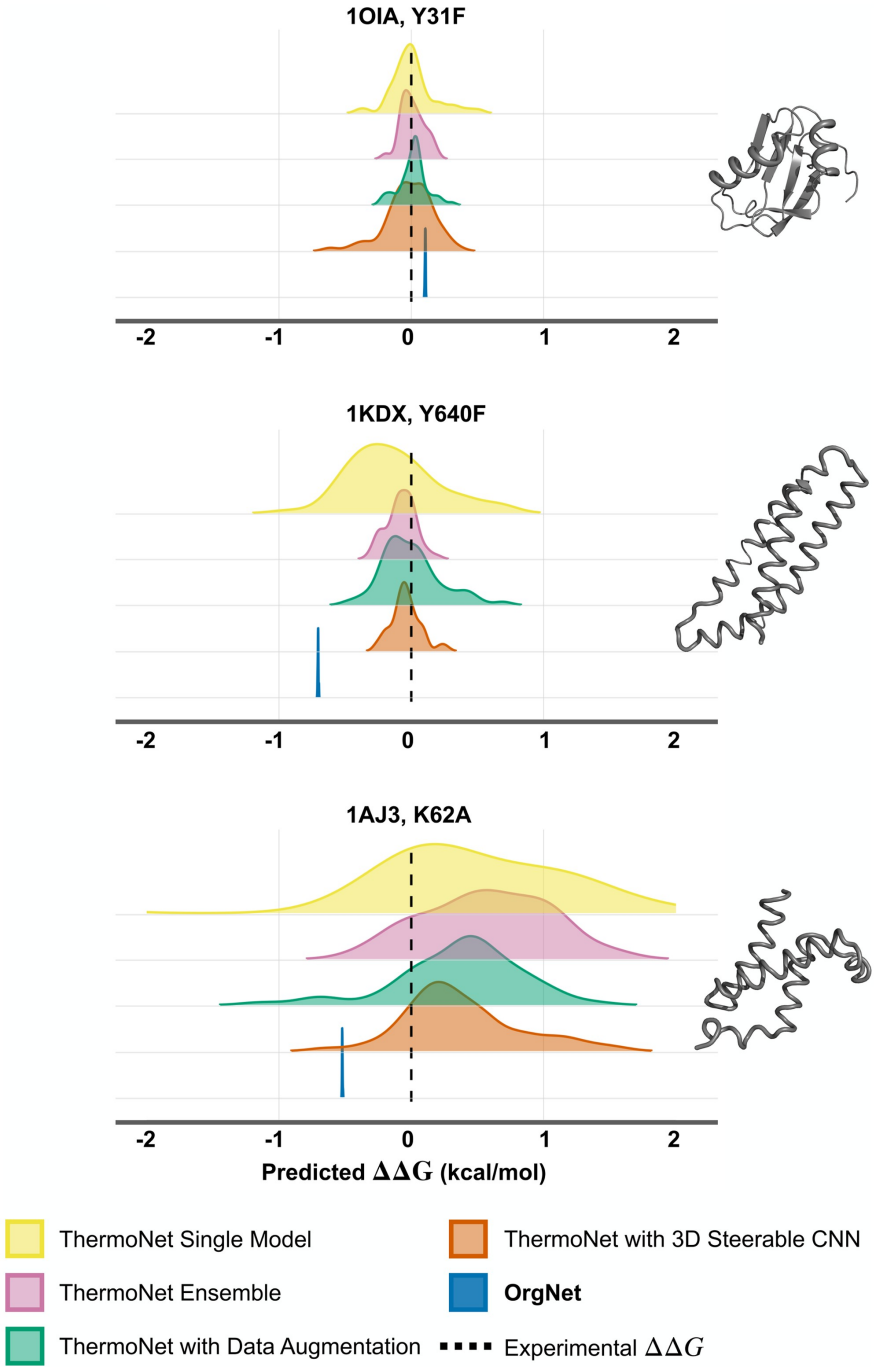
Illustration of the structure orientation bias problem in protein stability prediction. The ridge-line plots correspond to the distribution of the ΔΔG predictions computed with ThermoNet-based models using distinct orientations of the same structure. OrgNet predictions, shown in blue, are robust to orientation bias. Dashed vertical line corresponds to the experimental value of ΔΔG.

To highlight the problem of orientation bias in protein thermostability prediction, we examined ThermoNet (Li *et al.* 2020), one of the widely used structure-based methods for thermostability prediction, and applied it to different orientations of the same protein structures. Namely, the structures of alpha spectrin (PDB ID: 1AJ3, point mutation K26A), KIX domain of CREB binding protein (PDB ID: 1KDX, point mutation Y640F), and U1 small nuclear ribonucleoprotein A (PDB ID: 1OIA, point mutation Y31F) were rotated around 10 axes corresponding to the icosahedron vertices with a π/6 step, resulting in 120 distinct conformations. We then applied ThermoNet as well as the ensemble of ThermoNet models to each conformation to estimate the ΔΔ*G* value, and Fig. 1 shows the obtained results. As one can see, the predicted ΔΔ*G* values ranged from negative to positive values, and the difference can be as high as ∼6.0 kcal/mol (1AJ3 example). Remarkably, even the sign of the prediction, which is typically used as an indication of stabilizing or destabilizing effects, can vary with respect to the protein orientation. Therefore, input orientation strongly affects the prediction, thus limiting the reliability of the 3D CNN methods applied to protein thermostability prediction problem. To circumvent this problem, one can use data augmentation by providing the model with different orientations of the same sample during training. However, determining the optimal number of additional orientations is not straightforward. Moreover, this approach does not guarantee SE(3)-invariance. An alternative is to use an SE(3)-equivariant neural network architecture, such as 3D steerable CNNs (Weiler *et al.* 2018). However, such an approach operates with volumetric data, but orienting the protein structure and calculate voxels from it is, generally speaking, not the same as orienting voxel grids of the protein. Figure 1 shows the prediction distributions obtained with a ThermoNet model trained on the 10-times augmented dataset and the ThermoNet-like model with 3D CNN layers replaced to the 3D steerable CNN blocks. As one can see from the distributions, none of the approaches provide consistent predictions.

### 3.2 The OrgNet approach

**Figure 2.**
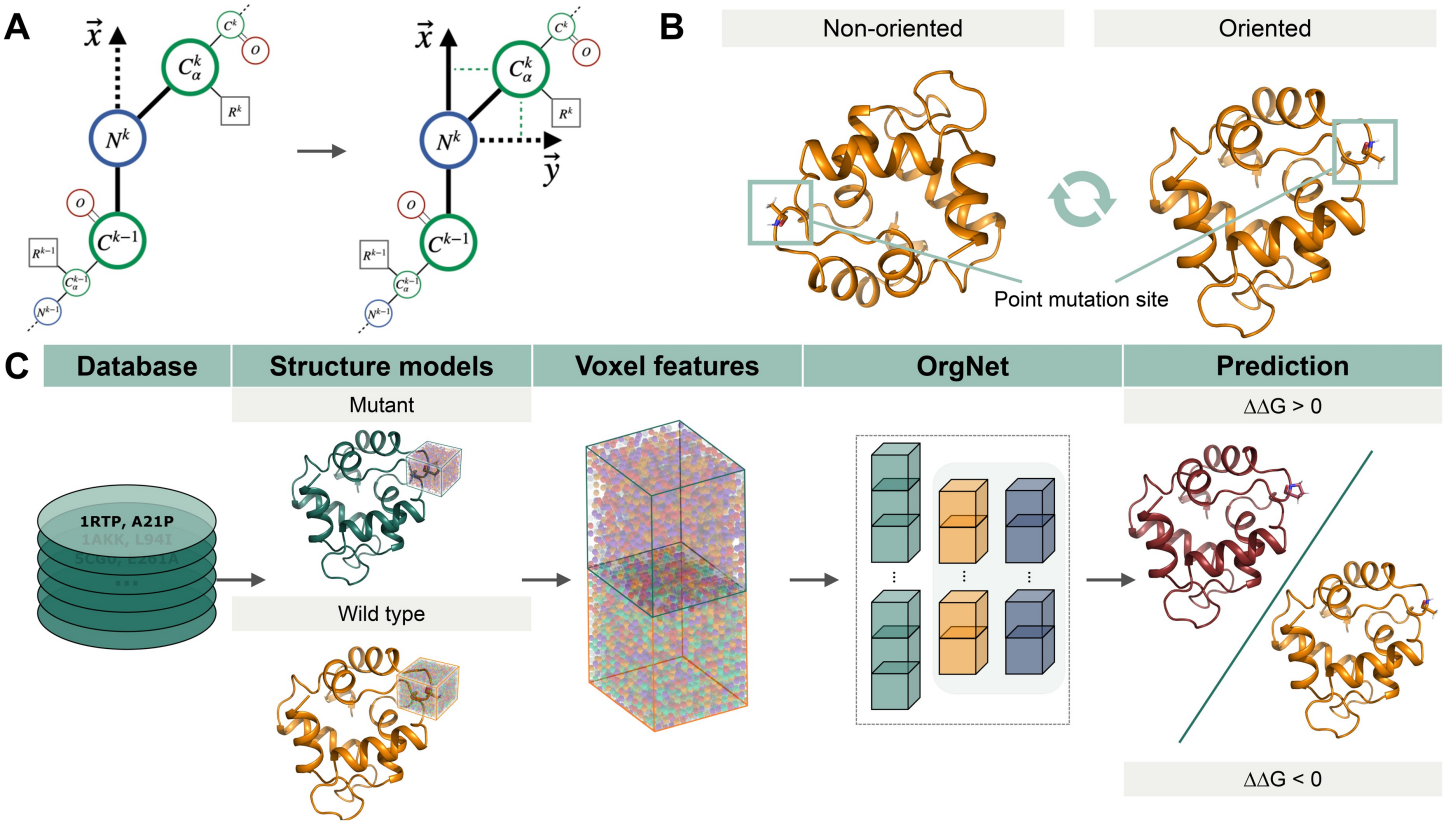
Illustration of the OrgNet workflow, consisting of protein orientation standardization, voxelization, model training, and prediction stages. (A) Schematic definition of the oriented coordinate frame. In the first step the *x*-axis is defined via direction of the $C^{k-1}-N^{k}$ bond. Then, the *y*-axis is defined in the $C^{k-1}-N^{k}-C_{\mathrm{alpha}}^{k}$ plane. Finally, the *z*-axis is defined via vector product of **x** and **y**. (B) The orientation standardization procedure aligns coordinate frames with respect to the point mutation site. (C) For the oriented wild-type and mutant-type structures in the dataset, voxel grids are computed and used for training. Each voxel grid comprises seven channels corresponding to physicochemical properties: hydrophobicity, hydrogen bond acceptor capacity, hydrogen bond donor capacity, aromaticity, positive ionizability, negative ionizability, and occupancy (Jiménez *et al.* 2017). The model output is the predicted ΔΔG value, indicating the stabilizing or destabilizing effect of a point mutation.

In this study, we aimed to enhance the performance of 3D CNN-based methods for protein thermostability prediction by leveraging the SE(3) variance of the 3D convolution operations. To achieve this, we introduced an orientation standardization of the input protein structures before computing the input encoding for 3D CNN. Specifically, we define a fixed coordinate frame coupled with basis vectors related to the backbone atoms of the substituted residue, determine spatial transform for the input orientation of the mutation site, and apply this transform to generate the standardized orientation of the input structure (see Methods and Fig. 2). The standardized orientation draws inspiration from the work of Pages and Grudinin (Pagès *et al.* 2019), where the authors aligned the input density maps corresponding to individual protein residues to derive protein quality assessment models. Such orientation allows one to eliminate the SE(3) variance problem of 3D CNNs because any input orientation is transformed into the standardized orientation.

To test whether standardizing input orientations improves the performance of the 3D CNN models, we first examined the architecture of ThermoNet, which demonstrated good performance on the test benchmarks (Li *et al.* 2020). For a rigorous comparison, we adopted the workflow described in Li *et al.* (2020), which involved preprocessing the training (Q1744, Q3214) and validation ($S^{\mathrm{sym}}$) datasets (Pucci *et al.* 2018) to generate structural models for mutant- and wild-type proteins using Rosetta (Kellogg *et al.* 2011) and voxel grids using HTMD (Doerr *et al.* 2016). The obtained voxel grids for a point mutation correspond to concatenated voxel grids computed for the wild-type and mutant-type structures. The voxel grid channels encompass seven properties: hydrophobicity, hydrogen bond acceptor capacity, hydrogen bond donor capacity, aromaticity, positive ionizability, negative ionizability, and occupancy (Jiménez *et al.* 2017). The dimensions of the computed voxel grids are [16, 16, 16, 7], where the first three dimensions correspond to (*x*, *y*, *z*) coordinates, and the last dimension corresponds to the seven properties. Consequently, the dimensions of the concatenated voxel grids that are fed to the 3D CNN as input are [16, 16, 16, 14]. The ThermoNet architecture consists of three 3D convolutional layers and one dense layer, producing an estimation of ΔΔ*G* for a given point mutation. We trained the ThermoNet-like models with and without the standardized orientation procedure, evaluated their performance on the $S^{\mathrm{sym}}$ test set. For comparison, we also considered the ThermoNet-like models trained using the augmented datasets and 3D steerable CNN layers (Weiler *et al.* 2018) (see Methods). The obtained results are summarized in Table 1. We observed that models trained with orientation standardization consistently outperformed ThermoNet models for both Q1744 and Q3214 datasets. Specifically, the mean absolute error (MAE), root mean square error (RMSE), and Pearson correlation coefficient (r) improved from 1.03 to 0.88, from 1.46 to 1.25, and from 0.54 to 0.66, respectively, for the Q3214 dataset. We have also observed an improvement in the performance metrics for the $S^{669}$ test set (see Supplementary Material, Supplementary Table S2). Moreover, the models trained with orientation standardization outperformed models trained using the augmented datasets as well as 3D steerable CNN layers. These results strongly indicate that removing orientation bias plays a significant role in improving the accuracy and reliability of 3D CNN-based point mutation stability prediction.

**Table 1.** Performance metrics on the $S^{\mathrm{sym}}$ test set for ThermoNet-like models trained using various approaches to reduce the SE(3)-variance including the orientation standardization procedure.^a^

Approach to reduce SE(3)-variance	Train	Direct			Reverse		
		MAE	RMSE	r	MAE	RMSE	r
No	q1744	1.14^b^	1.62^b^ (1.56)	0.42^b^ (**0.47**)	1.15^b^	1.63^b^ (1.55)	0.40^b^ (0.47)
Data augmentation	q1744	1.11	1.57	0.45	1.12	1.56	0.45
3D steerable CNN	q1744	1.24	1.72	0.35	1.19	1.66	0.36
Orientation standardization	q1744	**1.07**	**1.53**	**0.47**	**1.08**	**1.53**	**0.49**
No	q3214	1.03^b^	1.46^b^ (1.42)	0.54^b^ (0.58)	1.04^b^	1.47^b^ (1.38)	0.55^b^ (0.59)
Data augmentation	q3214	1.00	1.42	0.56	0.99	1.40	0.56
3D steerable CNN	q3214	1.06	1.51	0.49	1.02	1.42	0.55
Orientation standardization	q3214	**0.88**	**1.25**	**0.66**	**0.91**	**1.30**	**0.65**

a Each model represents an ensemble from 10-fold cross-validation. The best metric values are highlighted in bold.

b The obtained values correspond to the reproduced ThermoNet models, while the values in parentheses are sourced from Li *et al.* (2020).

In the next step, we searched for a more powerful architecture of 3D CNN using 5-fold cross-validation with a homology-based split of the S2648 dataset (Dehouck *et al.* 2009) (see Methods). Note that we also used different stacking of the voxel grids corresponding to the wild-type and the mutant-type compared to ThermoNet ([WT, WT-MUT] instead of [WT, MT]), as it demonstrated slightly better performance metrics. The resulting 3D CNN architecture of OrgNet consists of an input block with a 3D convolutional layer (16 channels), intermediate blocks with 3D convolutions (80 and 400 channels), and ReLU activations followed by max-pooling. The output block incorporates a 3D convolutional layer (512 channels) with ReLU, followed by a regression head with two fully connected layers (512 and 128) utilizing GELU activations to generate the predictions. For the final OrgNet model, we used an ensemble of five models derived from 5-fold cross-validation. As one can see from Fig. 1, OrgNet does provide consistent predictions across the different orientations of the protein structures.

### 3.3 Comparison with other methods

To compare OrgNet with other structure-based methods, we used the $S^{669}$ dataset (Pancotti *et al.* 2022) as the test dataset. Note that using S2648 as the train set and $S^{669}$ as the test set presents a more challenging setup compared to commonly used random train-test splits of combined datasets, as the $S^{669}$ dataset specifically comprises proteins with low homology to S2648. We compared the performance of OrgNet against other structure-based methods listed in Table 2. However, other methods used different training sets or training-validation strategies; therefore, a direct comparison of the performance metrics may not be straightforward. Nonetheless, OrgNet demonstrates competitive performance among other predictors. Notably, it demonstrates balanced performance for direct and reverse mutation prediction tasks: Pearson correlation coefficients (r) of 0.42 and 0.42, RMSE values of 1.55 and 1.56, and MAE values of 1.12 and 1.14, respectively. For comparison, another structure-based method, INPS3D, trained on the same homology-based splits as OrgNet, achieves similar performance metrics for the direct mutations but worse metrics for the reverse mutations, with correlation coefficients of 0.43 versus 0.33 and RMSE values of 1.50 versus 1.77, respectively. To assess the significance of the observed results, we performed the statistical test as it follows. For each pair of predictions, $y_{\text{OrgNet}}$ from OrgNet and $y_{\text{other}}$ from a different method, we assign “head” if $\left|y_{\text{true}}-y_{\text{OrgNet}}|<|y_{\text{true}}-y_{\text{other}}\right|$, and “tail” otherwise. The null hypothesis posits that the observed count of “heads” follows the distribution expected from random coin tosses. A binomial statistical test is then applied to test the null hypothesis. We found that OrgNet outperforms most other methods with statistical significance (*P*-value < 0.05), and performs comparably to DDGun3D and ACDC-NN (see Supplementary Material). Interestingly, the statistical significance primarily arises from the methods’ performance on reverse mutations. For direct mutations, however, the null hypothesis cannot be rejected.

**Table 2.** Performance metrics of OrgNet and other models on the $S^{669}$ dataset.^a^

Method	Method type	Train	Direct			Reverse		
			r	RMSE	MAE	r	RMSE	MAE
**OrgNet**	3D CNN	S2648	0.42	1.55	1.12	0.42	1.56	1.14
ACDC-NN (Benevenuta *et al.* 2021)	CNN	S2648 + Varibench	**0.46**	1.49	**1.05**	**0.45**	1.5	1.06
DDGun3D (Montanucci *et al.* 2019)	Linear parametric model	S2648^b^, Varibench	0.43	1.6	1.11	0.41	1.62	1.14
PremPS (Chen *et al.* 2020b)	RF	S2648	0.41	1.5	1.08	0.42	**1.49**	**1.05**
ThermoNet (Li *et al.* 2020)	3D CNN	Q1744	0.39	1.62	1.17	0.38	1.66	1.23
Rosetta (Kellogg *et al.* 2011)	Energy function	–	0.39	2.7	2.08	0.4	2.68	2.02
Dynamut (Rodrigues *et al.* 2018)	RF	S2648	0.41	1.6	1.19	0.34	1.69	1.24
INPS3D (Savojardo *et al.* 2016)	SVR	S2648	0.43	1.5	1.07	0.33	1.77	1.31
SDM (Worth *et al.* 2011)	Energy function	S2648^b^	0.41	1.67	1.26	0.13	2.16	1.64
PoPMuSiC 2.1 (Dehouck *et al.* 2011)	Linear parametric model	S2648	0.41	1.51	1.09	0.24	2.09	1.64
MAESTRO (Laimer *et al.* 2016)	LR, ANN and SVM	S2648	0.5	**1.44**	1.06	0.2	2.1	1.655
FoldX (Schymkowitz *et al.* 2005)	Empirical force field		0.22	2.3	1.56	0.22	2.48	1.5
DUET (Pires *et al.* 2014a)	SVM	S2648	0.41	1.52	1.1	0.23	2.14	1.68
I-Mutant3.0 (Capriotti *et al.* 2005)	SVM	S2648^c^	0.36	1.52	1.12	0.15	2.32	1.87
mCSM (Pires *et al.* 2014b)	Gaussian Regression and RF	S2648, S1925	0.36	1.54	1.13	0.22	2.3	1.86
Dynamut2 (Rodrigues *et al.* 2021)	RF	S2648	0.34	1.58	1.15	0.17	2.16	1.69

a Metrics for other models are sourced from Pancotti *et al.* (2022) and Umerenkov *et al.* (2023), with additional data retrieved from Sun *et al.* (2024). The best metric values are highlighted in bold.

b Used as the benchmark.

c 2087 distinct single mutations from ProTherm (Bava *et al.* 2004).

Another common test set is $S^{\mathrm{sym}}$ (Pucci *et al.* 2018). For direct comparison with ThermoNet, we retrained the OrgNet model on the Q3214 dataset (see Methods), and Table 3 presents the obtained results. We want to emphasize, that $S^{\mathrm{sym}}$ shares homology proteins with $S^{2648}$ as well as $Q^{3214}$ datasets, therefore one should interpret these performance metrics with caution. Nevertheless, the OrgNet models exhibited strong and well-balanced performance metrics, consistently outperforming other methods. Regarding the MAE performance metric, OrgNet trained on S2648 yields values of 0.92 and 0.93 for direct and reverse point mutations, respectively. Similarly, OrgNet trained on Q3214 shows values of 0.90 and 0.88 for direct and reverse point mutations. Unfortunately, the MAE values for the other methods were not provided (Li *et al.* 2020).

**Table 3.** Performance metrics of various methods on direct and reverse mutations of the $S^{\mathrm{Sym}}$ dataset.^a^

			Direct		Reverse	
Method	Method type	Train	RMSE	r	RMSE	r
**OrgNet**	3D CNN	S2648	1.31	0.61	1.33	0.60
**OrgNet**	3D CNN	Q3214	1.25	0.64	**1.23**	**0.64**
ThermoNet^b^ (Li *et al.* 2020)	3D CNN	Q3214	1.42	0.58	1.38	0.59
DDGun3DMontanucci *et al.* [2019]	Linear parametric model	S2648^b^, Varibench	1.42	0.56	1.46	0.53
ThermoNet (Li *et al.* 2020)	3D CNN	Q1744	1.56	0.47	1.55	0.47
PoPMuSiC^sym^ (Pucci *et al.* 2015)	Linear parametric model	S2648	1.58	0.48	1.62	0.48
MAESTRO (Laimer *et al.* 2016)	LR, SVM, NN	S2648	1.36	0.52	2.09	0.32
FoldX (Schymkowitz *et al.* 2005)	Empirical force field	–	1.56	0.63	2.13	0.39
PoPMuSiC 2.1 (Dehouck *et al.* 2011)	Linear parametric model	S2648	1.21	0.63	2.18	0.25
SDM (Worth *et al.* 2011)	Energy function	S2648^b^	1.74	0.51	2.28	0.32
iSTABLE (Chen *et al.* 2020a)	Meta-predictor	S3568	1.10	0.72	2.28	−0.08
I-Mutant 3.0 (Capriotti *et al.* 2005)	SVM	S2648^c^	1.23	0.62	2.32	−0.04
NeEMO (Giollo *et al.* 2014)	Neural Network	S2648	1.08	0.72	2.35	0.02
DUET (Pires *et al.* 2014a)	SVM	S2648	1.20	0.63	2.38	0.13
mCSM (Pires *et al.* 2014b)	Gaussian Regression and RF	S2648, S1925	1.23	0.61	2.43	0.14
MUPRO (Cheng *et al.* 2006)	SVM	S1615	**0.94**	**0.79**	2.51	0.07
STRUM (Quan *et al.* 2016)	Gradient Boosting regression	Q3421	1.05	0.75	2.51	−0.15
Rosetta (Kellogg *et al.* 2011)	Energy function	–	2.31	0.69	2.61	0.43
AUTOMUTE (Masso and Vaisman 2008)	SVM and RF	S1948, S1615, S1791, S1396, S2204	1.07	0.73	2.61	−0.01
CUPSAT (Parthiban *et al.* 2006)	Statistical method	4024^d^	1.71	0.39	2.88	0.05

a Metrics for other models and additional data are sourced from Li *et al.* (2020). The best metric values are highlighted in bold.

b Used as the benchmark.

c 2087 distinct single mutations from ProTherm (Bava *et al.* 2004).

d 4024 non-redundant protein structures obtained from the PISCES web server (Wang and Dunbrack Jr 2003).

Note that, in some cases, one may be interested in classifying point mutations as stabilizing or destabilizing rather than predicting ΔΔG values. We have trained classification OrgNet models by labeling point mutations as stabilizing (ΔΔG<0) or de-stabilizing (ΔΔG>0), and adjusting the output so that it corresponds to the probability of a point mutation being stabilizing. We observed that in terms of the classification performance metrics, OrgNet also demonstrates competitive performance (see Supplementary Material for the complete list of the metrics for OrgNet and other methods and Supplementary Fig. S3 for the F1 scores with respect to different probability threshold values).

To summarize, OrgNet is a 3D CNN-based approach that provides consistent predictions with respect to protein structure orientations. It demonstrates competitive performance compared to other protein stability prediction methods and can also be applied as a classification model. We would like to note, that while OrgNet successfully eliminates the SE(3)-variance problem, it does not account for flexible deformations in protein structures. In fact, we observed that for different conformations obtained through molecular dynamics simulations of a protein structure, the predictions can become inconsistent (see Supplementary Material, Supplementary Fig. S1). Therefore, we believe that developing more robust predictive models that incorporate flexibility is an important direction for future research in protein stability prediction.

## 4 Conclusion

In this study, we proposed standardizing the input orientations of point mutation sites to circumvent the SE(3) variance problem in 3D CNNs. The derived model, dubbed OrgNet, consistently outperformed the previously developed 3D CNN-based method, ThermoNet. Moreover, OrgNet demonstrated robust performance compared to other state-of-the-art structure-based methods. Specifically, it achieved one of the lowest root mean squared errors and exhibited balanced performance across both direct and reverse mutation prediction scenarios. These findings underscore the importance of addressing orientation bias in 3D CNNs and demonstrate that eliminating such bias yields more powerful models. Finally, we anticipate that integrating the proposed approach with complementary methods—such as sequence-based, evolution-based, LLM-based techniques—could lead to even more advanced predictive models.

## Supplementary Material

btaf252_Supplementary_Data

## Data Availability

The OrgNet models, along with the code for performing orientation standardization, calculating voxels, reproducing ThermoNet, and training ThermoNet-like models, are publicly available at https://github.com/i-Molecule/OrgNet. The datasets prepared in this study are publicly available on Zenodo (https://zenodo.org/). These datasets include the calculated voxels for both non-oriented and oriented datasets (Q1744, Q3214, S2648, $S^{669}$, $S^{\mathrm{Sym}}$), different orientations of the 1OIA, 1KDX, and 1AJ3 protein structures, as well as conformations from molecular dynamics simulations of these proteins. The Supplementary Material includes supplementary figures and tables, results of the statistical tests, and performance metrics for the classification models.
