# Supplementary material for: OrgNet: orientation-gnostic protein stability assessment using convolutional neural networks
Source: Bioinformatics. 2025 Jul 15;41(Suppl 1):i458–65. doi: 10.1093/bioinformatics/btaf252 (PMC12261421; doi:10.1093/bioinformatics/btaf252)
Supplement: btaf252_Supplementary_Data [file btaf252_supplementary_data.zip › btaf252_Supplementary_Data/Popov.5.sup.1.pdf]

Bioinformatics, 2025, pp. 1–2

doi:
ISMB/ECCB 2025, Conference Proceedings

ISMB/ECCB 2025, CONFERENCE PROCEEDINGS

Supplementary information

Ilya Buyanov,[1*] Anastasia Sarycheva[2,3,4*] and Petr Popov[2,3,4**]

[1]iMolecule, Skolkovo Institute of Science and Technology, Moscow, 121205, Russia, [2]Constructor Knowledge Institute, Bremen, 28759, Germany, [3]Constructor University Bremen gGmbH, Bremen, 28759, Germany and [4]Tetra D AG, Shaffhausen, 8200, Switzerland
[*]These authors contributed equally to this work.[**]Corresponding author. ppopov@constructor.university

Abstract

Supplementary information for OrgNet: Orientation-gnostic protein stability assessment using convolutional neural networks.

Table S1. Performance metrics of the original ThermoNet (`https://github.com/gersteinlab/ThermoNet`) and our implementation of ThermoNet trained on Q1744 for the S^{Sym} test set.

			Direct				Reverse			
**Model**	**Train**	**Validation**	**MAE**	**MSE**	**RMSE**	**r**	**MAE**	**MSE**	**RMSE**	**r**
reproduced	Q1744	S^{Sym}	1.14	2.61	1.62	0.42	1.15	2.65	1.63	0.40
original	Q1744	S^{Sym}	1.13	2.56	1.60	0.43	1.13	2.57	1.60	0.41

Table S2. Performance metrics on the $S669$ test set for ThermoNet-like models trained on Q1744 or Q3214 datasets with and without the orientation standardization procedure. Each model represents an ensemble from ten-fold cross-validation.

		Direct			Reverse		
**Orientation Standardization**	**Train**	**MAE**	**RMSE**	**r**	**MAE**	**RMSE**	**r**
no	Q1744	1.21	1.65	0.4	1.22	1.64	0.4
yes	Q1744	**1.16**	**1.58**	**0.41**	**1.16**	**1.58**	**0.42**
no	Q3214	1.19	1.62	0.39	1.21	1.64	0.4
yes	Q3214	**1.15**	**1.58**	**0.4**	**1.16**	**1.6**	**0.41**

© The Author 2025. Published by Oxford University Press. All rights reserved. For permissions, please e-mail: journals.permissions@oup.com

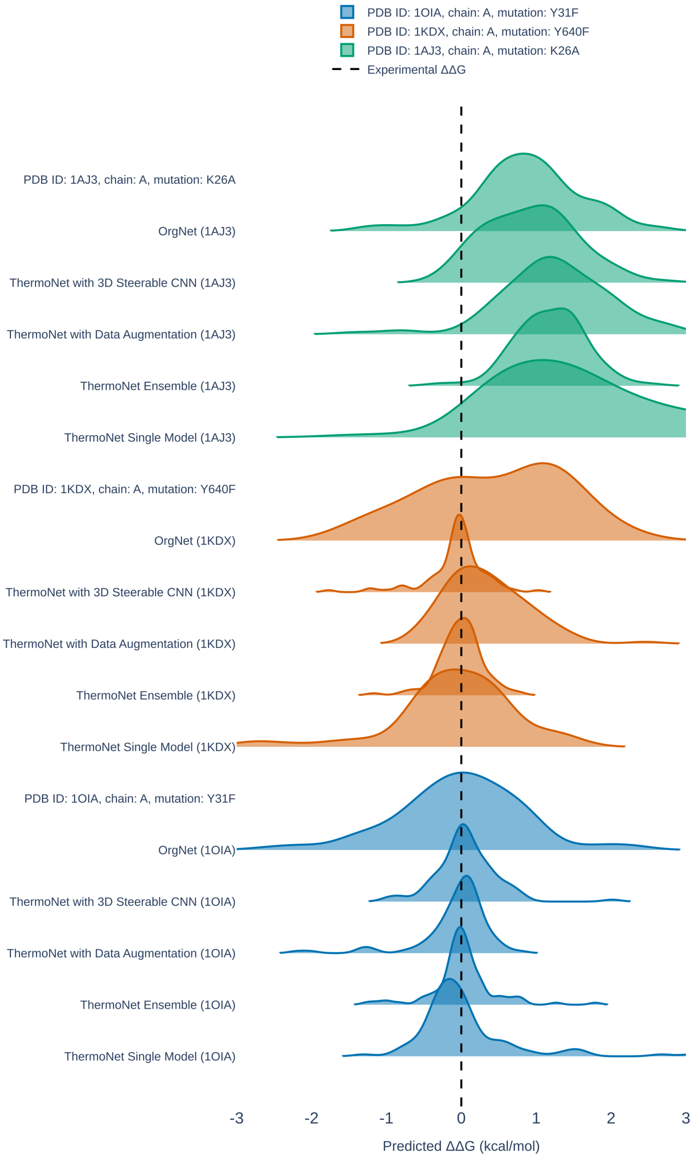

Fig. S1. Distribution of the $\Delta\Delta G$ predictions of ThermoNet-like models and OrgNet for the conformational ensembles obtained with 100ns-long full-atom molecular dynamics simulations of the protein structures (PDB IDs: 1AJ3, 1KDX, 1IOA).

Sequence-based similarity analysis of the datasets

Given a pair of datasets (D1 and D2), we have calculated the maximum sequence similarity for each sequence from D1 relative to D2 using a global pairwise alignment algorithm implemented in Biopython's pairwise2 module. Each alignment was performed using a simple scoring scheme in which matches were assigned a score of +1 and mismatches a score of 0 using the globalxx function. From the set of alignments generated, the top-scoring (first) alignment was selected for further analysis. Sequence similarity was then calculated as the percentage of aligned positions with identical amino acids with respect to the total lenght of the alignment. Figure S2 demonstrates the percentage of sequences in D1 that have at least one similar sequence in D2 with respect to the different similarity thresholds.

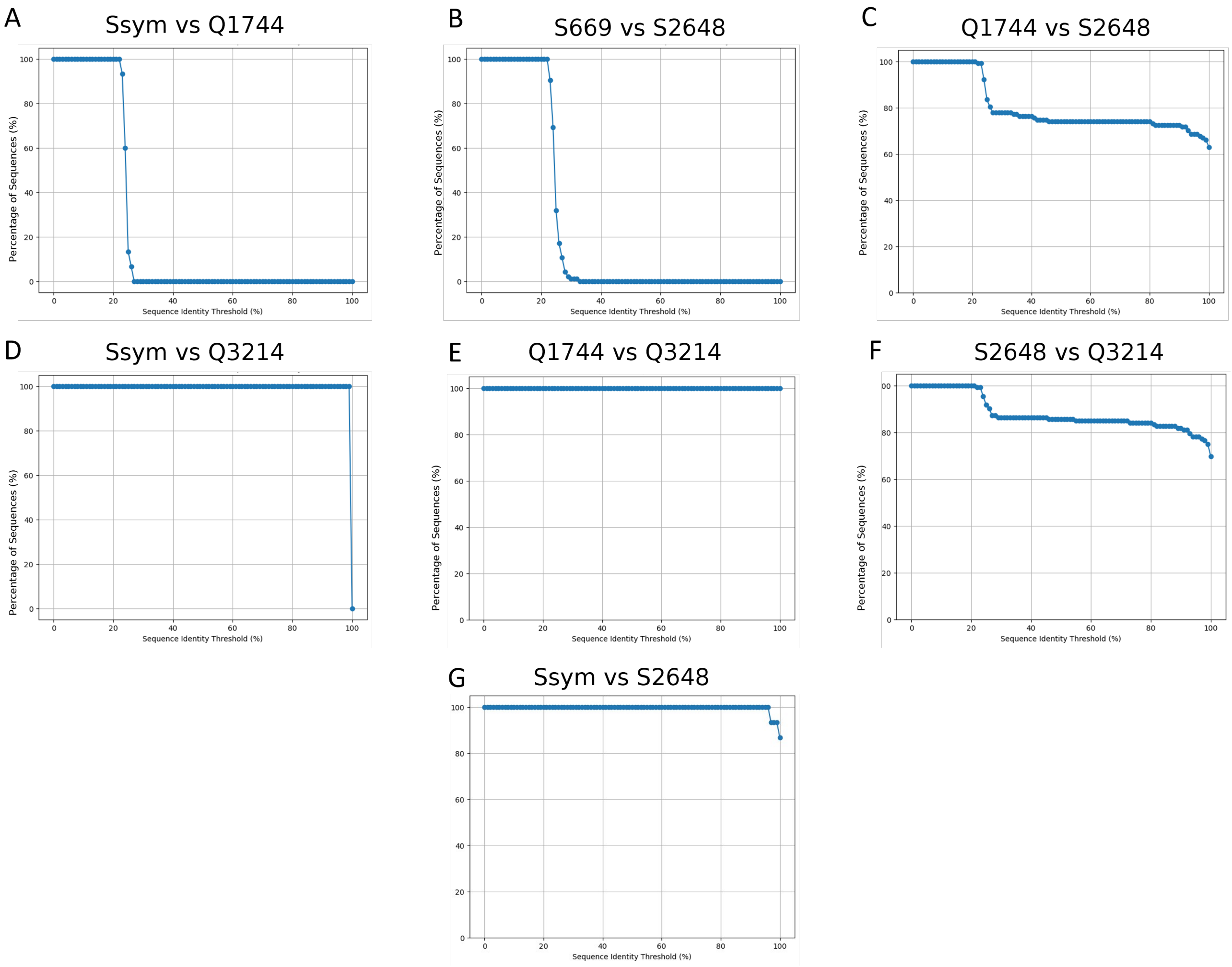

Fig. S2. Sequence-based similarity between different pairs of the datasets.

Table S3. Information about sequence-based similarity between the pairs of datasets. 'D' stands for dataset, 'p' - for protein, 's' - for sample, 'SS' - for sequence similarity.

#D1	#p in D1	#s in D1	#D2	#p in D2	#s in D2	#p SS >25%	#p SS>50%	#p SS>75%	#p SS=1	#identical p	#identical s
S669	94	669	S2648	132	2648	33	0	0	0	0	0
Ssym	15	342	Q3214	148	3214	15	15	15	14	12	1
Ssym	15	342	Q1744	127	1744	2	0	0	0	0	0
Q1744	127	1744	Q3214	148	3214	127	127	127	127	127	1744
Q1744	127	1744	S2648	132	3214	106	94	94	80	70	887
S2648	132	2648	Q3214	148	3214	121	113	111	92	82	1374
Ssym	15	342	S2648	132	2648	15	15	15	13	13	194

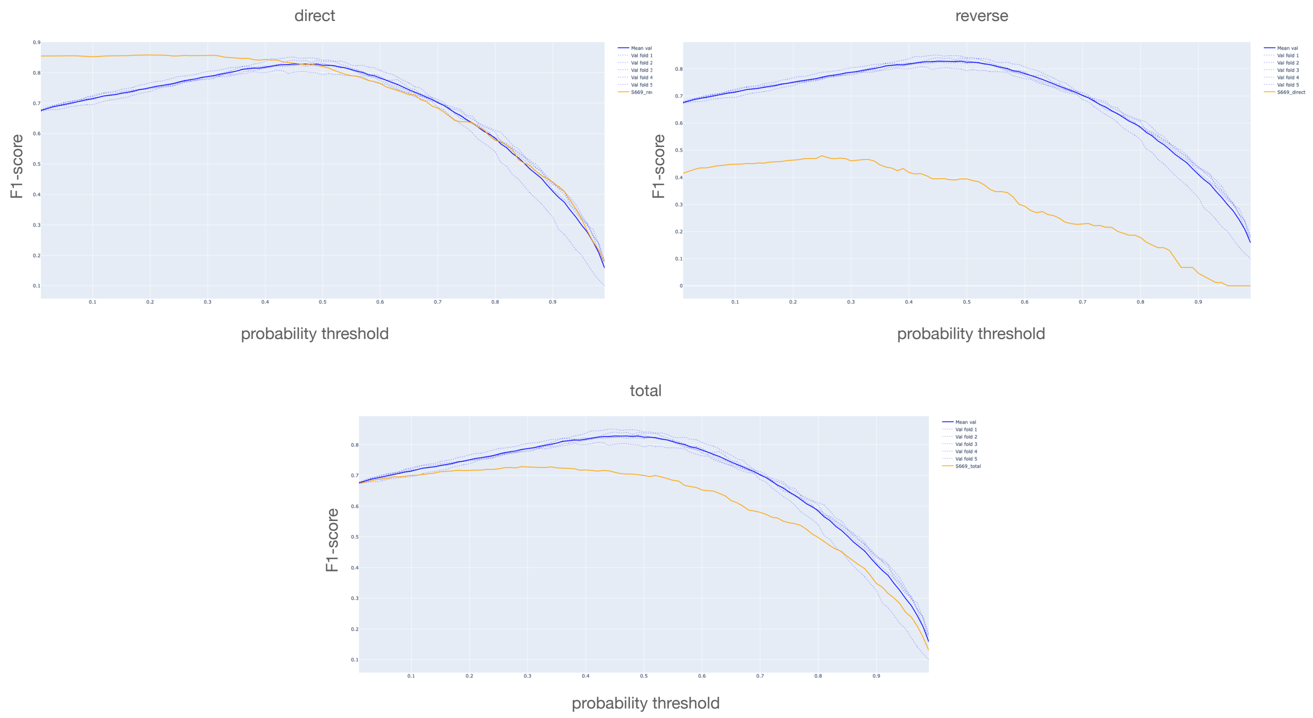

Fig. S3. The F1-score values with respect to the probability treshold values for the classification OrgNet models. The blue lines correspond to the validation scores (five validation folds of the S2648 dataset), and the orange line correspond to the test scores (S^{669} dataset).

Table S4. Point mutations for which the pipeline to calculate voxel grids failed.

Q1744	Q3214	S669	S2648
1pinA_K6A	1pinA_K6A	1diva_M1A	1aj3a_H10A
1aj3A_H10A	1aj3A_H10A	1diva_M1G	1aj3a_H10G
1yccA_K73W	1yccA_K73W	1guab_N56M	1cspa_M1R
1divA_M1G	1divA_M1G	2hbba_M1A	1ey0a_K6A
1yccA_K73A	1yccA_K73A	3dv0i_I130A	1ey0a_K6G
1yccA_K73G	1yccA_K73G	3dv0i_I130G	1huua_M69I
1divA_M1A	1divA_M1A	3dv0i_I130V	1luca_A81H
1aj3A_H10G	1aj3A_H10G	3dv0i_V129A	1lvea_L27CN
1arrA_M1A	1stnA_K6G	3dv0i_V129G	1lvea_L27CQ
1yccA_K73H	1bpiA_R1A		1lvea_V27BL
1yccA_K73L	1arrA_M1A		1lvea_Y27DD
1yccA_K73V	1lz1A_K1M		1lz1a_K1A
1cspA_M1R	1yccA_K73H		1lz1a_K1M
1yccA_K73R	1stnA_K6A		1ttqa_A18G
1yccA_K73I	1yccA_K73L		1ttqa_A18V
	1lz1A_K1A		1ttqa_F22I
	1yccA_K73V		1ttqa_F22L
	1cspA_M1R		1ttqa_F22V
	1yccA_K73R		1ttqa_Y175Q
	1yccA_K73I		1ttqa_L209V
			1ttqa_I232V
